# Impact of Virtual Reality Technology on Pain and Anxiety in Pediatric Burn Patients: A Systematic Review and Meta-Analysis

**DOI:** 10.3389/frvir.2021.751735

**Published:** 2022-01-06

**Authors:** Kathryn L. Smith, Yang Wang, Luana Colloca

**Affiliations:** 1 Department of Pain and Translational Symptom Science, School of Nursing, University of Maryland, Baltimore, MD, United States,; 2 Center to Advance Chronic Pain Research, University of Maryland, Baltimore, MD, United States,; 3 Departments of Anesthesiology and Psychiatry, School of Medicine, University of Maryland, Baltimore, MD, United States

**Keywords:** pediatrics, burn wound care, nonpharmacological intervention, acute pain management, distraction analgesia

## Abstract

**Introduction:**

Virtual reality (VR) has the potential to lessen pain and anxiety experienced by pediatric patients undergoing burn wound care procedures. Population-specific variables require novel technological application and thus, a systematic review among studies on its impact is warranted.

**Objective:**

The objective of this review was to evaluate the effectiveness of VR on pain in children with burn injuries undergoing wound care procedures.

**Methods:**

A systematic literature review was performed using PubMed and CINAHL databases from January 2010 to July 2021 with the keywords “pediatric,” “burn,” “virtual reality,” and “pain.” We included experimental studies of between- and within-subjects designs in which pediatric patients’ exposure to virtual reality technology during burn wound care functioned as the intervention of interest. Two researchers independently performed the literature search, made judgements of inclusion/exclusion based on agreed-upon criteria, abstracted data, and assessed quality of evidence using a standardized appraisal tool. A meta-analysis was conducted to evaluate the effectiveness of the VR on burning procedural pain in pediatric population. Standardized mean difference (SMD) was used as an index of combined effect size, and a random effect model was used for meta-analysis.

**Results:**

Ten articles published between January 2010 and July 2021 passed the selection criteria: six randomized controlled trials and four randomized repeated-measures studies. Consistent results among the studies provided support for VR as effective in reducing pain and potentially pain related anxiety in children undergoing burn wound care through preprocedural preparation (n = 2) and procedural intervention (n = 8). VR effects on pain intensity ratings were moderate to large (SMD=0.60, 95%CI=0.28–0.93, *p*=0.0037 with no significant heterogeneity of VR intervention effects between studies. Only one study reported direct influence of VR intervention on pre-procedural situational anxiety with a moderate effect size (Cohen’s d = 0.575, 95%CI = 0.11–1.04).

**Conclusion:**

Children’s exposure to VR during burn care procedures was associated with lower levels of pain and pain related anxiety. Moderate to large effect sizes support the integration of VR into traditional pediatric burn pain protocols irrespective of innovative delivery methods and content required for use in burned pediatric patients.

## INTRODUCTION

### The Problem of Pain Related to Burn Injuries in Children

Worldwide epidemiological research demonstrates the high prevalence of childhood burns, disproportionately greater in infants of ethnic minorities in developing nations (Alnababtah et al., 2016). Burn wounds require frequent debridement and dressing changes to heal appropriately with protection against subsequent infections, since the body’s natural barrier to infection is impaired. Burn victims experience excruciating nociceptive pain from damaged tissue, which is then followed, and often intensified, by procedural pain during routine wound care. Burned children often regard wound dressing changes as “the most traumatizing and frightening part of their experience of having a burn” because of the painful, observable nature of the procedure (McGarry et al., 2014). Concurrent anxiety also generates a challenging cycle in the management of pediatric burn patients. A child may become anxious in the anticipation of future pain, which, in turn, exacerbates the pain experienced during the actual procedure.

The current pharmacologic medications used to treat burn pain in children include high-dose opioid analgesics and benzodiazepines, anxiolytics, nonsteroidal anti-inflammatory, non-opioid, and sedative adjuncts. In addition to causing short-term respiratory and gastrointestinal effects, current pharmacologic interventions have been cited as insufficient in treating childhood burn-related pain and threaten the development of long-term tolerance (Melzack and Wall, 1965; de Jong et al., 2014). McMurtry et al. (2015) warn that unrelieved childhood pain may contribute to later phobias, leading to healthcare avoidance behaviors across the lifespan (McMurtry et al., 2015).

### Non-pharmacological Distraction

Based on Melzack and Wall (1965) gate control theory, an individual’s perception of procedural burn pain is dampened by the diversion of attention away from the wound care procedure and towards more pleasant amusements (Melzack and Wall, 1965). With a fixed amount of attentional load, a distracted patient has less resources available for pain perception. Psychological anxiety is also targeted with distraction. Playing cards, music, and balloon inflation have all been documented as effective diversion methods in significantly reducing children’s pain and anxiety perception during unpleasant medical procedures (Sahiner and Bal, 2016).

Virtual reality (VR) technology may improve a child’s burn wound care experience better than other distraction techniques by capturing more attentional load through interactive, immersive, and multi-sensory characteristics (Won et al., 2017). VR aims to transport its user into an alternate environment as “a distraction method that provides the user with real-time interaction with computer-simulated entities in a pseudo-natural immersion via multisensory stimulation” (Won et al., 2017). Immersion, interaction, and navigation in this virtual world modulates pain and anxiety awareness to generate an analgesic result (Hoffman et al., 2004; Gutierrez-Martinez et al., 2010). In fact, studies have examined the potential of VR in reducing both acute (Kipping et al., 2012; Colloca et al., 2020) and chronic pain (Darnall et al., 2020) from experimental environment to clinical settings. Those studies often used distinct VR contexts with different levels of immersion and interaction, which may influence the VR effects in alleviating pain.

### Special Considerations in Pediatric Burn Population

Systematic reviews emphasize VR’s promising effect on procedural pain and anxiety in adult patients with burn injuries (Scapin et al., 2018), however, conventional VR software content may not be appropriate for the pediatric-aged population, especially considering that most pediatric burns and hospitalizations occur in children one to 4 years of age (Peck et al., 2011). A recent systematic review and meta-analysis studied the effect of VR on pain reductions experienced during general medical procedures without a focus on burn population (Eijlers et al., 2019). It should be noted that traditional headsets and handheld controllers used in virtual reality delivery restrict children with burns on their face and upper extremities from use (Dahlquist et al., 2008; Dumoulin et al., 2019; Gerçeker et al., 2021). Burn wound debridement may also involve water, in some cases a child’s wound may be fully submerged within a hydrotank during therapy, and thus, it is crucial for the VR equipment to be water-resistant (Khadra et al., 2018). When considering ethical research practices, the VR technology should be able to be used in tandem with pharmacologic and other non-pharmacologic interventions.

The population- and disease-specific characteristics of pediatric burn patients undergoing wound care procedures require novel applications of VR technology to address pain and pain related anxiety. The current systemic review and meta-analysis sought to assess the effectiveness of VR (when compared to standard care) in reducing pain and anxiety experienced by children during burn wound care procedures across different software content, delivery methods, and interaction immersion designs.

## METHODS

This review was conducted following the Preferred Reporting Items for Systematic Reviews and Meta-Analyses (PRISMA (Page et al., 2021)) guidelines.

### Search Strategy

The literature search was conducted in July 2021 using PubMed and CINAHL databases. The details search terms combinations were included in Table 1. The search was conducted using the Boolean operator “OR” to include the possible pediatric terms (“Pediatric” OR “Child” OR “Kid” OR “Minor” OR “Youth” OR “Teen*” OR “Adolescent” OR “School-Age” OR “Toddler” OR “Infant”), possible virtual reality terms (“Virtual reality” OR “Virtual immersion” OR “Virtual reality game” OR “Virtual distraction” OR “Virtual reality technology”), and possible pain related terms (“Pain” OR “Anxiety” OR “Distress” OR “Stress” OR “Procedural” OR “Acute” OR “Discomfort” OR “Fear” OR “Hurt”). Possible pediatric terms, burn terms (“Burn”), virtual reality terms, and pain related outcome terms were combined with Boolean operator “AND” for final search input.

### Selection Inclusion and Exclusion Criteria

The following criteria for inclusion/exclusion in the current review were established:

1. Research design: between- or within-subjects primary experiments. Both design types deemed acceptable given valuable insights gathered through both designs. Direct comparisons between VR exposure and standard of care were able to be drawn from between-subjects designed studies. Within-subjects designed studies allowed for control of individual patient factors (i.e., temperament, preprocedural analgesia, caregiver present). Study protocols, reviews, conference papers, abstracts, dissertations and case studies were excluded.
2. Population: pediatric patients under the age of 18 years undergoing burn wound care procedure (i.e., debridement, dressing change). Study populations of both adult and pediatric patients were excluded. Rehabilitation-focused procedures (i.e., post-injury physical therapy) were excluded.
3. Intervention: exposure to VR defined by technology’s goal to engage, immerse, distract patient in virtual environment. Witmer and Singer (1998) traditional definition of VR as an environment displayed through a head-mounted device was expanded given population- and burn injury-specific requirements (Witmer and Singer, 1998). To minimizing the risk of contaminations, patients with burn injuries may not be able to wear a head-mounted device. Therefore, no limitations were placed on content or immersion strategy of VR world. The type of VR used in pediatric burn context were reported in the result section.
4. Outcomes: A quantitative measure of pain. *Self-reported measure* of pain given by pediatric patient was the primary outcome. The results from two studies (Khadra et al., 2018; Khadra et al., 2020) were included in the review but later excluded from meta-analysis because of their lack of a patient self-report pain outcome. All other studies featured a patient self-report measure of pain, and thus, direct comparisons were able to to made through the meta-analysis with eight of the 10 total studies. *Observational pain ratings* from parent, caregiver, and healthcare clinician were also included in the review. Anxiety was the secondary outcome of interest due to its influence on pain, however, studies deficient of anxiety measures were not excluded.
5. Publication type and language: Full text, peer-reviewed articles published in academic journals written in English language.
6. Publication date: Published within the last 10 years due to VR technological advances and applicability to current practice environment.

### Study Selection

The search results were independently screened by researchers (K.L.S. and Y. W.). Discrepancies in inclusion judgments were discussed as a group (K.L.S., Y.W., L.C.) until consensus was met.

### Data Abstraction and Evidence Quality Appraisal

The primary outcome of the current meta-analysis was the self-reported pain intensity. The secondary outcome was the self-reported anxiety level. Data points pertaining to the following variables were systematically abstracted from each study and put into Table 2: study participants, sample age range (in years), sample size, study design, intervention, control group(s) with or without randomization technique, self-reported pain outcome with measure, caregiver’s observational pain outcome with measure. The effect sizes for self-reported pain intensity outcome variables were later included in Table 2 for easy comparison between studies. The level and quality of evidence collected in each study was rated based on the Johns Hopkins Nursing Evidence-Based Practice Research Evidence Appraisal Tool for later qualitative consideration (Newhouse et al., 2007).

### Meta-Analysis

Meta-analysis was conducted for the primary outcome self-reported pain ratings. There was only one paper reporting the effect of VR on pre-procedural anxiety. Thus, meta-analysis was omitted for the secondary outcome self-reported anxiety. A random effects model was adopted to conduct a meta-analysis to meet the review’s objective of evaluating the effect of novel VR technology on pediatric burn pain across studies with multiple VR styles.

The standardized mean difference Cohen’s *d* was adopted as an index for effect size. For studies that did not report effect sizes, we calculated Cohen’s *d* for individual studies following (Lakens, 2013). In particular, for between-subjects studies, Cohen’s *d* was calculated as d=(Mean_2_-Mean_1_)/SD_pooled_, where Mean_2_ and Mean_1_ represented the average score from the control group and the VR group, respectively. SD_pooled_ was calculated as $\sqrt{\frac{\left(n_{1}-1\right)SD_{1}^{2}+\left(n_{2}-1\right)SD_{2}^{2}}{n_{1}+n_{2}-2}}$. For within-subjects studies, Cohen’s d was calculated as $\text{d}=\frac{{\text{Mean}}_{2}-{\text{Mean}}_{1}}{\sqrt{SD_{1}^{2}+SD_{2}^{2}-2\times r\times SD_{1}\times SD_{2}}}\times \sqrt{2\left(1-r\right)}$ where r represented the correlations between VR condition and the control conditions. For studies that did not report correlations, a correlation r = 0.5 was used following (Borenstein et al., 2010).

Random effects model was used to calculate the combined effect size (Borenstein et al., 2010). 95% confidence interval (CI) was reported for the combined effect size. To test the potential heterogeneity of the effect sizes between studies, we performed chi-square test and calculated the I^2^ values. A significant chi-square test indicated significant heterogeneity among the included studies. I^2^ was used to quantify the amount of the heterogeneity. Following (Higgins et al., 2003), I^2^ values of 25%, 50% and 75% were considered to be low, moderate and high heterogeneity. Whenever significant heterogeneity was observed, subgroup analysis was designed to conduct with the type of VR (multi-model distraction vs. projector based VR vs. interactive based VR) treating as subgroups, for the purpose of detecting possible source of heterogeneity.

### Sensitivity Analysis

In order to verify if the findings from the meta-analysis were biased by low-quality studies and/or individual studies with large effect sizes, sensitivity analysis was conducted by re-calculating combined effect sizes after removing the low quality studies (Quality B and C) and large effect sizes studies. Similar to the main analysis, a random effect model was used for the sensitivity analysis.

## RESULTS

### Study Characteristics

**FIGURE 1 |:**
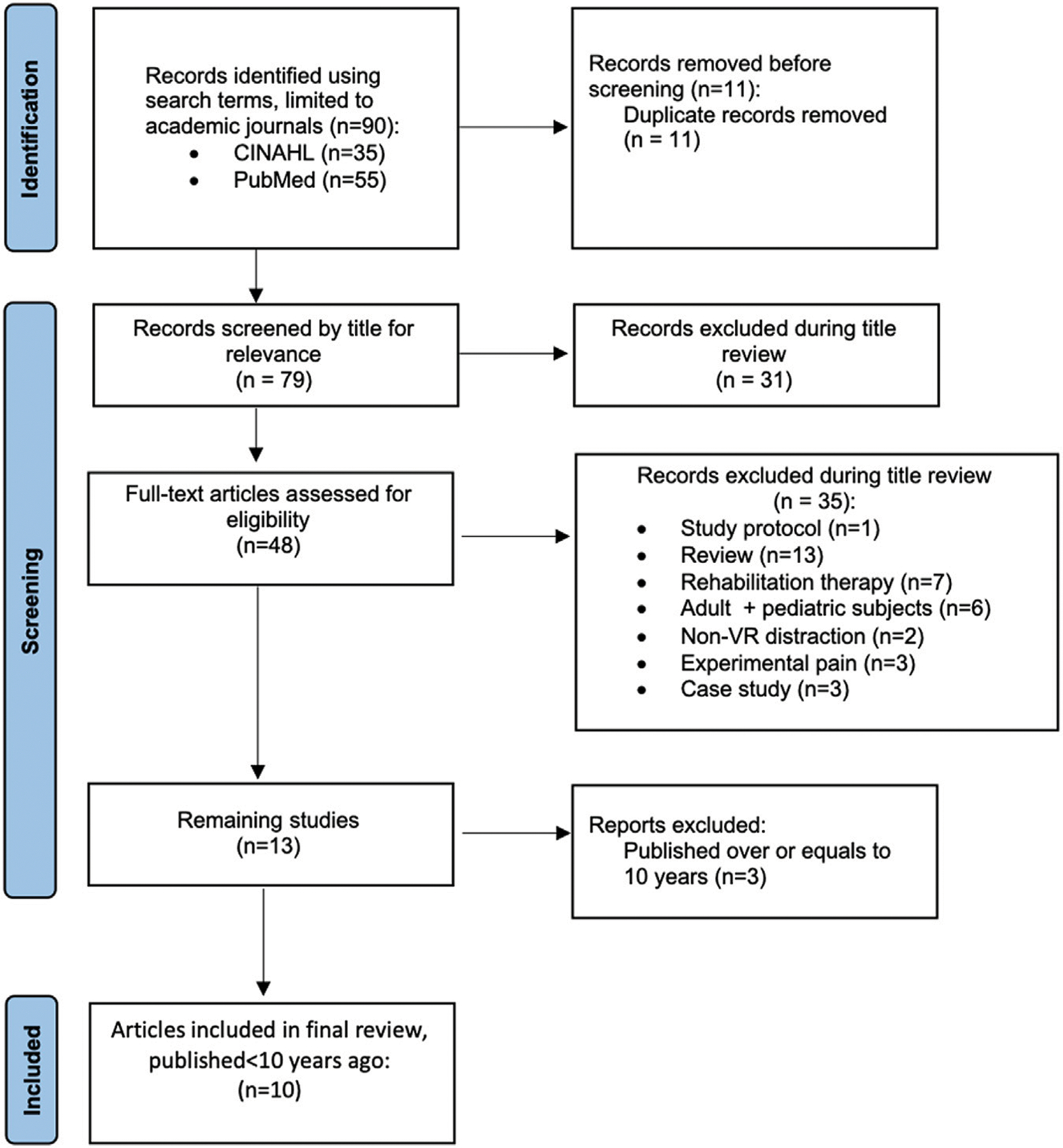
Study selection process. 90 publications in the literature were identified using the search terms. After the screening and full-text reading, the current review included 10 relevant publications.

The initial literature search yielded 90 individual articles, of which ten were included in the final study pool, resulting in a final sample size of 445 children with burn injuries (See Figure 1 for a detailed flow chart of the search process and selection process). The characteristics of the individual studies were detailed in Table 2. The ten studied were published within the last 10 years from 2011 to 2021. Six studies featured a between-subjects design with two separate groups of participants, one group exposed to VR and the other group not exposed to VR. The other four studies featured a within-subjects design with the same participants exposed to both VR and non-VR conditions. Assignment into VR versus non-VR groups and order of exposure to VR versus non-VR conditions was usually randomized or counterbalanced. Sample sizes varied from 15 to 90 participants, with the within-subjects designed studied having smaller sample sizes presumably from higher statistical power of within-subjects designs. International use of VR as a potential resource for pediatric burn injuries have been examined across different countries including United States of America (4 studies), Canada (3 studies) and Australia (3 studies).

### Sample Populations

The final dataset resulted in 445 participants with burn injuries. Distinct age ranges were observed across the 10 selected studies. Children of preschool to teen ages made up the sample in five out of the ten studies, while two studies purposively included infants and toddlers up to school-age children to capture a unique subset of younger pediatric patients. The samples in the Brown et al. (2014) and Miller et al. (2011) studies include preschoolers to young teenagers, with age ranges of 4–13 years and 3–10 years respectively (Miller et al., 2011; Brown et al., 2014). Children falling within a wider range of 6–17 years were included in the Hoffman et al. (2019, 2020) studies, most of which were from Latin American (Hoffman et al., 2019; Hoffman et al., 2020). Adolescent-aged patients comprised the comparison groups in Kipping et al. (2012), Jeffs et al. (2014), and Le May et al. (2021) studies (Kipping et al., 2012; Jeffs et al., 2014; Le May et al., 2021). Khadra et al. (2018, 2020) chose to focus primarily on infants and toddlers recruited from a hospital in Canada; the mean age of children of those two studies were around 2.2 years with a 3-month-old as the youngest participant in the study cohort (Khadra et al., 2018; Khadra et al., 2020). Future research is needed to understand how pediatric age may impact the effectiveness of VR in pediatric populations.

### VR Intervention and Exposure

The current review identified variations of VR technology, in both software contents and delivery methods, across all studies. Thus, results obtained in each of the studies are limited in terms of generalizability.

#### Multi-modal distraction.

In Brown et al. (2014) and Miller et al. (2011), children interacted with the “Bobby Gets a Burn” preprocedural preparation story before their wound care (Miller et al., 2011; Brown et al., 2014). This device allowed participant to interact with the virtual environment and get auditory, visual and vibration feedbacks by movement and touching the screen. However, this device did not provide an immersive experience because the visual stimulation was provided by a 2-D tablet.

#### Projector-based VR.

Khadra et al. (2018, 2020) were the only two studied that adopted a projector-based VR for infants or children with burn injuries (Khadra et al., 2018; Khadra et al., 2020). The projector-based VR was set up via a curved screen in front of the patients, a projector and a remote controller with which the participants could change the component of the VR games such as speed of the object and the visual angles. The advantage of projector-based VR was to create an immersive environment without wearing a headset to minimize possible contaminations. It should be noted that those two studies were excluded when performing meta-analysis in order to reduce bias in the data-analysis and result interpretation. Observation pain ratings emphasized the pain distress perspective as compared to the self-reported ratings (Manne et al., 1992). Given that observational ratings relied on caregivers observing pain behaviors such as crying, facial grimaces, arms, and legs movements, it was an indirect assessment of pain experience (Cohen et al., 2008). Therefore, we excluded those two studies when calculating the combined effect sizes.

#### Interactive VR.

Interactive VR refers to the VR games that involves interactions of the patients to the virtual environment. In Hoffman et al. (2019, 2020) and Jeffs et al. (2014) studies, *VR Snow World* was adopted and found to be effective in alleviating pain during the wound care (Jeffs et al., 2014; Hoffman et al., 2019; Hoffman et al., 2020). The snowy VR environment was designed with special considerations given to pediatric burn patients. It created an environment of snow world and the participant was able to interact with the snow man in the scenery. To avoid unnecessary head movements for children with burn injuries, the VR was provided on a tripod arm mount instead of head-mounted-display. The snowy scenery was adopted to illicit a possible cold analgesic association. Kipping et al. (2012) used similar mounted articulated-arm and provided two VR context *Chicken Little*^*™*^ and *Need for Speed* which also involved interactive components (Kipping et al., 2012). Unlike the above mentioned studies which employed a mounted articulated arm to present VR, Le May et al. (2021) used head-mounted-display device to increase the level of immersion. The interactive video game DREAMLAND was used for participants during the burning care (Le May et al., 2021).

Special care should be taken to interpret study results applicably, as better analgesia likely results from VR environments that promote presence through interaction and sensory feedback. It should be noted that VR systems featured across the studies required interactions through a computer mouse, joystick controller, or trackball; perhaps, a comparison in VR efficacy between external controller versus head-movement control is worthwhile.

### Non VR-Intervention/Comparison Groups

#### Standard care.

The majority of the included studies employed standard pain medication care as a comparison group/exposure (Khadra et al., 2018; Hoffman et al., 2019; Hoffman et al., 2020; Khadra et al., 2020; Le May et al., 2021).

#### Standard distraction.

Three out of ten studies adopted standard distraction as a comparison group/condition to be compared with VR. Standard distraction methods including television, stories, music, and caregiver support were available to patients assigned to the standard distraction (SD) group. Patient’s choices were not recorded limiting the ability to draw clear comparisons in pain outcome measures between VR and standard distraction group participants.

#### Passive distraction.

Jeffs et al. (2014) used a passive distraction comparator whereas participants assigned to passive distraction watched the “Cloudy with a Chance of Meatballs” movie on an arm-mounted television (Jeffs et al., 2014). Little description was provided for the conditions presented to patients in the standard care group, however, it is assumed that burn care was provided by the nurse as usual without explicit distraction methods provided.

#### Passive VR.

Xiang et al. (2021) used a passive VR condition where participants watched a virtual reality context using the head-mounted-display without the joysticks, so that the interactive component was removed from the passive VR condition (Xiang et al., 2021).

### Outcomes Measures

Many different measures of pain and distress are featured throughout the studies included in the current review, of which self-reports of pain on the VAS and nurses’ scores of distress on Faces, Legs, Activity, Cry, Consolability (FLACC) assessment are the most common. In addition to the subjective measurement of pain and anxiety, objective measurement including heart rate and oxygen saturations have been recorded as physiologic markers to capture a more compressive picture of pain. Featured in the study conducted by Jeffs and others (2014), data collected on the Spielberg State-Trait Anxiety Inventory for Children supports the relationship between pain and anxiety (Jeffs et al., 2014). In future studies, it is valuable to include other measures of situational and state anxiety and temperament to increase knowledge regarding individual factors related to pain experiences and associated distress.

#### Patient Self-Reported Outcomes

Brown et al. (2014) and Miller et al. (2011) adopted multi-modal distraction (MMD) as VR interventions. Faces Pain Scale-R (FACES) were used to assess pain intensity ratings. Brown et al. (2014) found that on the third change of pediatric study dressing, during the dressing removal, children in the standard group indicated an average of 2.43 pain intensity on the Faces Pain Scale-Revised (FPS-R) versus an average of 0.73 pain intensity reported by children in the MMD group. Similarly, across all pain measures, Miller et al. (2011) found that children in the MMD group showed reduced levels of pain than children in the standard care group with significant differences observed between pain intensity (*p* < 0.001) and distress behaviors (*p* < 0.001) on the Wong Baker Faces and FLACC measures. The pain intensity scores reported by patients on the Wong Baker Faces scale indicated a significant difference between standard distraction (mean = 2.39, SD = 1.09) and MMD (mean = 0.7, SD = 0.86) groups.

In terms of active VR, in Kipping et al. (2012), although not statistically significant, differences observed on VAS between groups demonstrate a lower pain intensity experience by children in the active VR group (mean = 4.2, SD = 3.2) than in the standard distraction group (mean = 2.9, SD = 2.3) at time of dressing removal, indicative of a small effect size. Similar results were observed from Le May et al. (2021) which compared active VR and standard care using a within-subjects design. Children participants demonstrated significantly lower pain intensity and lower pain-related fear during active VR in compared with standard care.

Jeffs et al. (2014) featured a novel pain assessment tool, the Adolescent Pediatric Pain Tool with Word Graphic Rating Scale (APPT-WGRS), which included a body outline to identify pain location, a 100-mm line to visually mark felt pain intensity, and a word descriptor list for patients to pick sensory, affective, and evaluative pain qualities. The researchers report reliability and validity support of this measure through several other studies. Analyses run on the VAS scale data revealed that each unit-millimeter increase on the APPT in preprocedural pain was associated with a 0.9-mm increase in procedural pain. Overall, less procedural pain was seen in adolescents in the VR group than the passive distraction group with a large pair-wise effect size between VR-PD groups. Interestingly, more procedural pain was observed in participants across groups treated with opioid medications. This piece of evidence provides further supports the need for non-pharmacological complementary strategies to current medication pain management measures.

#### Caregiver Observational Report Pain Outcomes

Khadra et al. (2018, 2020) were the two studies that reported only caregiver observational pain outcomes (Khadra et al., 2018; Khadra et al., 2020). Pain assessments were made using FLACC scores documented by nursing staff at five different points throughout the procedure: 1 h pre-procedure, on arrival at the hydrotherapy tank room, 10 min after beginning procedure concurrent with debridement, immediately after the procedure before leaving the hydrotherapy room, and 30 min post-procedure. Non-significant differences in FLACC pain scores were observed before, during, and after the procedure (*p* = 0.264). However, average pain scores remained low throughout the procedure (Mean = 2.9). A bimodal distribution of FLACC pain scores during debridement was found; FLACC scores were either low (0–3/10) or severe (7–10/10). Satisfied, positive feedback from healthcare professionals further reinforced their conclusion.

#### Anxiety Outcomes

In terms of pain-related anxiety, Brown et al. (2014) assessed situational anxiety on a visual analog scale (VAS). Reduced anxiety levels before the dressing removal were found in the MMD group as compared to the non-VR group (Brown et al., 2014). Moreover, the reduction of anxiety levels also paralleled with a significantly lower maximum heart rates in VR group participants across all dressing changes when adjusted for age. Another study (Jeffs et al., 2014) used the Spielberger State-Trait Anxiety Inventory for Children to score individual’s transitory feelings of anxiousness (state anxiety) and usual nervousness level (trait anxiety). Given the relationship between anxiety and pain, this measure allows for a closer look at individual factors and possibility of influence on perceived pain. Their results provided support for a moderate correlation between state anxiety and preprocedural pain, meaning that adolescents with higher levels of distress before a wound care procedure experienced more preprocedural pain.

#### Wound Healing

Two studies examined how VR would facilitate the wound healing (Miller et al., 2011; Brown et al., 2014). Brown et al. (2014) assessed the re-epithelization rates as an index of wound healing. They found that the burn wounds of participants in the VR group healed on average 2.14 days faster than wounds of participants assigned to the non-VR group. This difference in re-epithelization rates was found to be statistically significant when adjusted for burn depth. Fewer total dressing changes were required in the VR group suggesting resource-saving potential. Similarly, Miller et al. (2014) showed that partial thickness burns of MDD participants healed on average 3 days faster than standard care participants, 15 versus 18 days.

### Meta-Analysis

**FIGURE 2 |:**
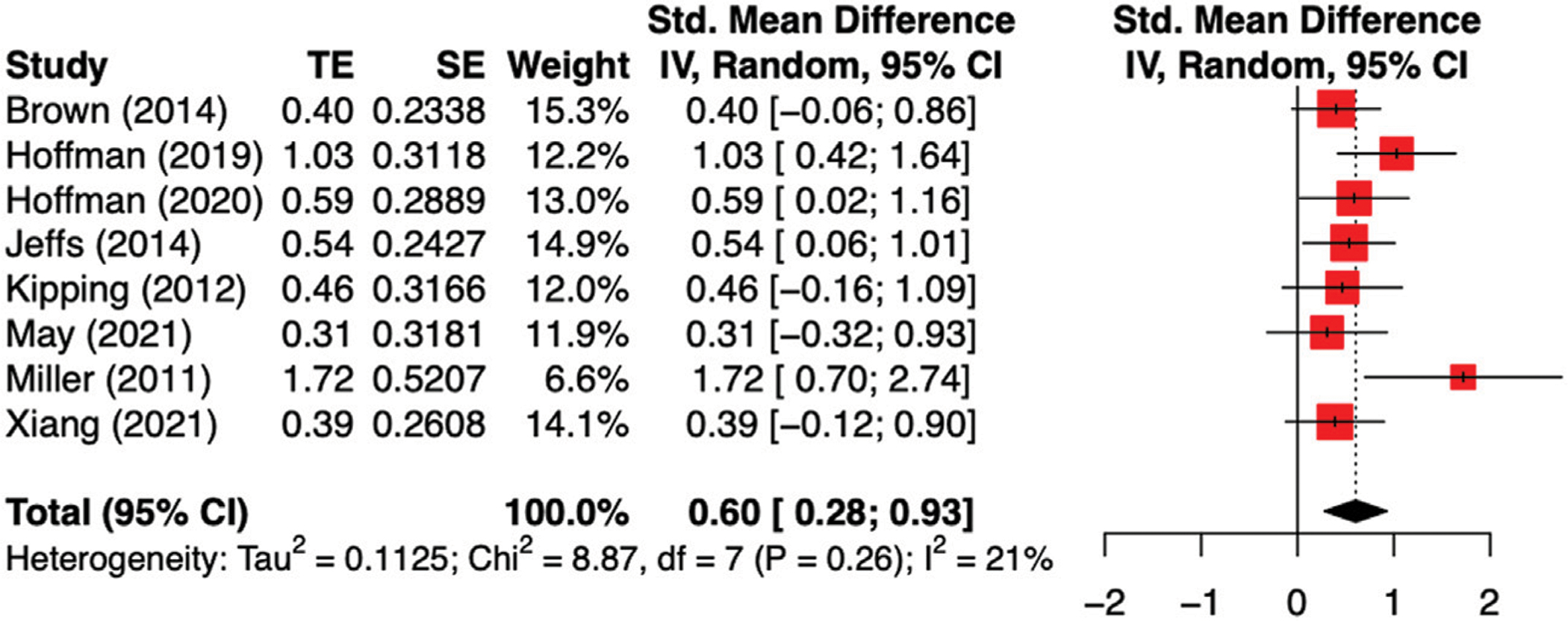
Met-analysis of the effect of VR on self-reported pain intensity during the wound care. The combined effect size was moderate Cohen’s d = 0.60 with non-significant between study heterogeneity.

Regarding the primary outcome self-reported pain ratings during the wound care, the meta-analysis revealed a moderate and significant combined effect size Standardized mean difference (SMD) when comparing VR to the standard care/distraction condition (SMD = 0.60, *p* = 0.0037, 95%CI = 0.28–0.90, Figure 2). The heterogeneity between studies were small and not significant, as revealed by the non-significant Chi-square test (Chi-square = 8.87, *p* = 0.26, I^2^ = 21%). Given that the heterogeneity was not significant across the included studies, no subgroup analysis was conducted.

When looked into different VR interventions, active VR yields medium to large effect sizes ranging from Cohen’s *d* of 0.39 (Xiang et al., 2021) to 1.03 (Hoffman et al., 2019), which was similar to the studies using Multi-Modal Distraction (Cohen’s *d* ranging from 0.4 to 1.72). VR was found to be effective in reducing self-reported pain when compared to standard distraction (e.g., TV, videos, books, and toys (Miller et al., 2011; Kipping et al., 2012; Brown et al., 2014)), as well as standard medication care (Hoffman et al., 2019; Hoffman et al., 2020).

In terms of the secondary outcome self-reported anxiety level, only one study (Brown et al., 2014) examined the effectiveness of VR on the pre-procedural situational anxiety with a moderate effect size (Cohen’s d = 0.575, 95%CI = 0.11–1.04).

### Sensitivity Analysis

After removing the low methodological quality studies and large effect size studies (Jeffs et al., 2014; Kipping et al., 2012; Miller et al., 2011), the overall effect size of VR on self-reported pain ratings remained significant. The combined effect size was moderate (SMD = 0.53, *p* = 0.013, 95%CI = 0.19–0.87), and was similar to the whole sample overall effect size (SMD = 0.60, *p* = 0.004, 95%CI = 0.28–0.90), suggesting a robust effectiveness of VR on burn pain during wound care in pediatric population.

## DISCUSSION

This review outlines an important arena for clinical practice for the potential to add an efficacious intervention in the arsenals of treatments for procedural pain and distress in children undergoing burn wound care procedures. The high-quality empirical support gathered by Brown et al. (2014) and Miller et al. (2011) for the efficacy of the MDD device in providing pain and situational anxiety relief for pediatric burn patients underscores the advantageous influence of preprocedural preparation (Miller et al., 2011; Brown et al., 2014). Squires (1995) reports that children experience stress derived from five distinct aspects of hospitalization: 1) new people, places, and routines, 2) unfamiliar food, clothing, and types of play, 3) “private part” exposure to strangers, 4) medical jargon, 5) pain and shameful feelings, and 6) observed anxiety of caregivers (Squires, 1995). Preprocedural preparation through VR devices familiarizes children with the healthcare professionals, procedural environment, and sequence of procedural events related to burn wound care reducing the overall situational foreignness (Justus et al., 2006). Caregivers are provided with a tangible tool to aid children through the procedural experience, and thus, their own nerves are eased. Preprocedural preparation should be incorporated into VR intervention protocols, along with procedural distraction.

Three out of the ten studies separated participants into a VR intervention group and standard distraction control group, in which a diverse array of different distraction interventions including television, video games, plastic toys, stories, and music were accessible to patients for use during their procedure (Miller et al., 2011; Kipping et al., 2012; Brown et al., 2014). It is likely that the efficacy of these different distraction options varied in degree of provided distress relief, and thus, it is more difficult to draw definitive conclusions regarding differences between the two study groups. Although insufficiently powered, the study conducted by Jeffs et al. (2014) splits participants into three study groups (VR, passive movie distraction, standard care) allowing for stronger deductions to be made about the specific effects of interventions provided (Jeffs et al., 2014). Expanding Jeffs et al. (2014) results, Xiang et al. (2021) compared active VR versus passive VR versus standard care in reducing pediatric burn pain with a greater sample size (n = 90) and found that children with burn injuries had lower worst pain when exposing to active VR that involves an interaction as compared to passive VR and the standard care, while passive VR and the standard care did not differ from one another in influencing the worst pain levels during the wound care (Xiang et al., 2021).

The six studies of parallel-group design limit the potential for altered expectancies across VR and non-VR conditions, meaning patients’ subjective experience of procedural pain is less likely to be altered by past experience with both treatment conditions. The benefit of a within-subject designed study, like the Hoffman et al. (2019) study, is that differences in individual factors are better controlled (Hoffman et al., 2019). By interacting with both VR and non-VR conditions, patients are better able to report interventional effects. Given the wide-ranging responses to pharmacologic medications between individuals and even wound care procedural days, it is important to eliminate in future research as much external “noise” as possible through experimental design to ascertain true VR-related outcomes.

### Strengths and Limitations

Measures that go beyond a mere collection of pain intensity, other dimensions of pain are worthwhile to explore (i.e., pain unpleasantness, mood changes, situational anxiety). Future research should also include a more comprehensive report of children’s past medical histories to assess the potential to include priory-experienced pain and traumatic events that can become a target to apply VR to minimize such experiences. Importantly, the issue of blinding commonly cited throughout the research, should be carefully addressed having different staff members to administer VR and collect post-procedural data assessments. Also, future research should include adequate VR approaches to control for placebo responses (e.g., sham VR tools, see (Honzel et al., 2019).

Although generalizability limitations prevent definitive recommendations pertaining to the implementation of other VR programs and delivery methods from being made, all studies included in this systematic review indicate the promising nature of VR interventions in improving pain-related outcomes in pediatric burn patients with the potential to also reduce anxiety and trauma required for better physical and psychological healing.

## CONCLUSION

Various VR inventions including interactive VR, projector-based VR and multi-modal distractions have been examined in the context of pediatric burn injuries procedural pain. Overall, there was significant and moderate effect size for VR induced analgesic effect (Cohen’s d = 0.60) during the burn wound care for children patients when compared to both standard distraction (e.g., TV, videos, books, and toys, as well as standard medication care. Moreover, different type of VR interventions did not seem to influence the magnitude of the analgesic effects as revealed by the non-significant heterogeneity of the included studies. Future research was needed to provide further evidence in supporting the effects of VR intervention on situational anxiety during the wound care in pediatric population.

## Supplementary Material

Suppl Materials

## Figures and Tables

**TABLE 1 |:** Search key terms.

S1	S2	S3	S4
Pediatric	Burn	Virtual reality	Pain
Child	-	Virtual immersion	Anxiety
Kid	-	Virtual reality game	Distress
Minor	-	Virtual distraction	Stress
Youth	-	Virtual reality technology	Procedural
Teen*	-	-	Acute
Adolescent	-	-	Discomfort
School-Age	-	-	Fear
Toddler	-	-	Hurt
Infant	-	-	-

S1, S2, S3, S4 search terms combined with “AND” for final search input.

**TABLE 2 |:** Descriptive information from the studies on the effect of VR on pediatric burn pain.

References	Participants	Sample age range (years)	Sample size	Study design	Intervention	Control Group(s)	Self-reported pain	Caregivers observational pain report	Effect size for self-reported pain intensity outcome (Cohen’s d)^a^	Level and quality rating of evidence
Brown et al. (2014)	Children with burn injuries in Australian pediatric burn clinic	4 to 13	n = 75, 35 for Ditto, 40 for standard distraction	Between-subjects	Ditto	Standard passive distraction (TV, videos, books, toys, parental soothing) (random assignment)	Pain intensity measured by the Faces Pain Scale-Revised (FPS-R)	Nurses observational pain measured by the Faces, Legs, Arms, Cry. Consolability (FLACC) scale	0.4	Level: I Quality: A
Hoffman et al. (2019)	Children with extensive, severe injuries in Latin American intensive care hospital unit	6 to 17	n = 48	Within-subjects	VR Snow World	Standard pain medication (order counterbalanced)	“Worst” (sensory) pain intensity, “unpleasantness” (affective) pain, and “time spent thinking about” (cognitive) pain measured by the Graphic Rating Scale (GRS)	-	1.03	Level: I Quality: A
Hoffman et al. (2020)	Large severe burn injuries children	6 to 17	n = 50, 25 for VR, 25 for standard pain medication	Between-subjects	VR Snow World	Standard pain medication	Worst pain intensity, pain unpleasantness, and time spent thinking about pain measured by the Graphic Rating Scale (GRS)	-	0.59	Level: I Quality: A
Jeffs et al. (2014)	Adolescents with burn injuries	10 to 17	n = 28; 8 for VR, 10 for passive distraction, and 10 for Standard care	Between-subjects	VR	Passive distraction, Standard care	Pain intensity, sensory, affective, and evaluative qualities of pain measured by the Adolescent Pediatric Pain Tool (APPT)	-	0.54	Level: I Quality: B
Khadra et al. (2018)	Children with burn injuries undergoing inpatient and outpatient hydrotherapy	0.2 to 10	n = 15	Within-subjects	Projector-based VR	Standard care (order counterbalanced)	-	Nurses observational pain measured by the Faces, Legs, Arms, Cry. Consolability (FLACC) scale	-	Level: II Quality: C
Khadra et al. (2020)	Burn injuries children	0.5 to 7	n = 38	Within-subjects	Projector-based VR	Standard care (order counterbalanced)	-	Nurses observational pain measured by the Faces, Legs, Arms, Cry. Consolability (FLACC) scale, and Numerical Rating Scale-obs (NRS-obs)	-	Level: II Quality: B
Kipping et al. (2012)	Adolescents with burn injuries in outpatient burn clinic	11 to 17	n = 41,20 for VR, 21 for Standard distraction	Between-subjects	VR	Standard distraction (TV, stories, music available)	Pain intensity measured by the Visual Analog Scale (VAS)	Nurses observational pain measured by the Faces, Legs, Arms, Cry. Consolability (FLACC) scale, and adolescent pain (VAS)	0.46	Level: I Quality: B
Le May et al. (2021)	Burn and fracture injuries children	7 to 17	n = 20	Within-subjects	VR	Standard care	Pain intensity measured by the Numerical Rating Scale (NRS)	Nurses observational comfort measured by OCCEB-BECCO	0.31	Level: II Quality: A
Miller et al. (2011)	Children with burn injuries	3 to 10	n = 40, 20 for Multi-modal distraction, 20 for standard distraction	Between-subjects	Multi-modal Distraction Ditto	Standard distraction (TV, stories, age-appropriate toys, nursing staff soothing) (randomization	Pain intensity measured by Wong Baker Faces Scale (FACES)	Nurses observational pain measured by the Faces, Legs, Arms, Cry. Consolability (FLACC) scale	1.72	Level: I Quality: A
Xiang et al. (2021)	Burn injuries children	6 to 17	n = 90, 31 for active VR, 30 for passive VR, 29 for standard care	Between-subjects	Active VR	Passive VR, Standard care	Pain intensity measured by Visual Analog Scale (VAS)	Researcher observational pain measured by the FLACC-revised	0.39	Level: I Quality: A

a Effect sizes were calculated for the self-reported pain only. Positive values for Cohen’s d indicate a higher value in the control group than in the treatment group. For measures evaluating pain, a positive Cohen’s d reflects a decrease in pain in the treatment group compared to the control.

Cohen’s d: | 0.2 | = small effect size | 0.5 | = medium effect size | 0.8 | = large effect size.

## References

[R1] Alnababtah K, Khan S, and Ashford R (2016). Socio-Demographic Factors and the Prevalence of burns in Children: An Overview of the Literature. Paediatrics Int. Child Health 36, 45–51. doi:10.1179/2046905514y.0000000157 25309999

[R2] Borenstein M, Hedges LV, Higgins JPT, and Rothstein HR (2010). A Basic Introduction to Fixed-Effect and Random-Effects Models for Meta-Analysis. Res. Synth. Method 1, 97–111. doi:10.1002/jrsm.12 26061376

[R3] Brown NJ, Kimble RM, Rodger S, Ware RS, and Cuttle L (2014). Play and Heal: Randomized Controlled Trial of Ditto Intervention Efficacy on Improving Re-epithelialization in Pediatric burns. Burns 40, 204–213. doi:10.1016/j.burns.2013.11.024 24360745

[R4] Cohen LL, Lemanek K, Blount RL, Dahlquist LM, Lim CS, Palermo TM, (2008). Evidence-Based Assessment of Pediatric Pain. J. Pediatr. Psychol 33, 939–955. doi:10.1093/jpepsy/jsm103 18024983 PMC2639489

[R5] Colloca L, Raghuraman N, Wang Y, Akintola T, Brawn-Cinani B, Colloca G, (2020). Virtual Reality: Physiological and Behavioral Mechanisms to Increase Individual Pain Tolerance Limits. Pain 161, 2010–2021. doi:10.1097/j.pain.0000000000001900 32345915 PMC7584744

[R6] Dahlquist LM, Weiss KE, Dillinger Clendaniel L, Law EF, Ackerman CS, and Mckenna KD (2008). Effects of Videogame Distraction Using a Virtual Reality Type Head-Mounted Display Helmet on Cold Pressor Pain in Children. J. Pediatr. Psychol 34, 574–584. 18367495 10.1093/jpepsy/jsn023 PMC2722134

[R7] Darnall BD, Krishnamurthy P, Tsuei J, and Minor JD (2020). Self-Administered Skills-Based Virtual Reality Intervention for Chronic Pain: Randomized Controlled Pilot Study. JMIR Form Res. 4, e17293. doi:10.2196/17293 32374272 PMC7381022

[R8] de Jong AEE, Bremer M, Van Komen R, Vanbrabant L, Schuurmans M, Middelkoop E, (2014). Pain in Young Children with Burns: Extent, Course and Influencing Factors. Burns 40, 38–47. doi:10.1016/j.burns.2013.09.017 24188991

[R9] Dumoulin S, Bouchard S, Ellis J, Lavoie KL, Vézina M-P, Charbonneau P, (2019). A Randomized Controlled Trial on the Use of Virtual Reality for Needle-Related Procedures in Children and Adolescents in the Emergency Department. Games Health J. 8, 285–293. 31135178 10.1089/g4h.2018.0111

[R10] Eijlers R, Utens EMWJ, Staals LM, De Nijs PFA, Berghmans JM, Wijnen RMH, (2019). Systematic Review and Meta-Analysis of Virtual Reality in Pediatrics. Anesth. Analgesia 129, 1344–1353. doi:10.1213/ane.0000000000004165 PMC6791566 31136330

[R11] Gerçeker GÖ, Bektaş M, Aydinok Y, Ören H, Ellidokuz H, and Olgun N (2021). The Effect of Virtual Reality on Pain, Fear, and Anxiety During Access of a Port with Huber Needle in Pediatric Hematology-Oncology Patients: Randomized Controlled Trial. Eur. J. Oncol. Nurs 50, 101886. doi:10.1111/j.1469-7580.2011.01469.x 33321461

[R12] Gutierrez-Martinez O, Gutierrez-Maldonado J, Cabas-Hoyos K, and Loreto D (2010). The Illusion of Presence Influences VR Distraction: Effects on Cold-Pressor Pain. Stud. Health Technol. Inform 154, 155–159. doi:10.3233/978-1-60750-561-7-155 20543289

[R13] Higgins JPT, Thompson SG, Deeks JJ, and Altman DG (2003). Measuring Inconsistency in Meta-Analyses. BMJ 327, 557–560. doi:10.1136/bmj.327.7414.557 12958120 PMC192859

[R14] Hoffman HG, Patterson DR, Rodriguez RA, Peña R, Beck W, and Meyer WJ (2020). Virtual Reality Analgesia for Children with Large Severe Burn Wounds during Burn Wound Debridement. Front. Virtual Real 1, 602299. doi:10.3389/frvir.2020.602299 33585833 PMC7880045

[R15] Hoffman HG, Rodriguez RA, Gonzalez M, Bernardy M, Peña R, Beck W, (2019). Immersive Virtual Reality as an Adjunctive Non-Opioid Analgesic for Pre-Dominantly Latin American Children with Large Severe Burn Wounds during Burn Wound Cleaning in the Intensive Care Unit: A Pilot Study. Front. Hum. Neurosci 13, 262. doi:10.3389/fnhum.2019.00262 31440148 PMC6694842

[R16] Hoffman HG, Sharar SR, Coda B, Everett JJ, Ciol M, Richards T, (2004). Manipulating Presence Influences the Magnitude of Virtual Reality Analgesia. Pain 111, 162–168. doi:10.1016/j.pain.2004.06.013 15327820

[R17] Honzel E, Murthi S, Brawn-Cinani B, Colloca G, Kier C, Varshney A, (2019). Virtual Reality, Music, and Pain: Developing the Premise for an Interdisciplinary Approach to Pain Management. Pain 160, 1909–1919. doi:10.1097/j.pain.0000000000001539 30817437 PMC7279616

[R18] Jeffs D, Dorman D, Brown S, Files A, Graves T, Kirk E, (2014). Effect of Virtual Reality on Adolescent Pain during Burn Wound Care. J. Burn Care Res 35, 395–408. doi:10.1097/bcr.0000000000000019 24823326

[R19] Justus R, Wyles D, Wilson J, Rode D, Walther V, and Lim-Sulit N (2006). Preparing Children and Families for Surgery: Mount Sinai’s Multidisciplinary Perspective. Pediatr. Nurs 32, 35–43. 16572537

[R20] Khadra C, Ballard A, Déry J, Paquin D, Fortin J-S, Perreault I, (2018). Projector-Based Virtual Reality Dome Environment for Procedural Pain and Anxiety in Young Children with Burn Injuries: A Pilot Study. J. pain Res 11, 343–353. doi:10.2147/jpr.s151084 29491717 PMC5817417

[R21] Khadra C, Ballard A, Paquin D, Cotes-Turpin C, Hoffman HG, Perreault I, (2020). Effects of a Projector-Based Hybrid Virtual Reality on Pain in Young Children with Burn Injuries during Hydrotherapy Sessions: A Within-Subject Randomized Crossover Trial. Burns 46, 1571–1584. doi:10.1016/j.burns.2020.04.006 32389349

[R22] Kipping B, Rodger S, Miller K, and Kimble RM (2012). Virtual Reality for Acute Pain Reduction in Adolescents Undergoing Burn Wound Care: A Prospective Randomized Controlled Trial. Burns 38, 650–657. doi:10.1016/j.burns.2011.11.010 22348801

[R23] Lakens D (2013). Calculating and Reporting Effect Sizes to Facilitate Cumulative Science: A Practical Primer for T-Tests and ANOVAs. Front. Psychol 4, 863. doi:10.3389/fpsyg.2013.00863 24324449 PMC3840331

[R24] Le May S, Hupin M, Khadra C, Ballard A, Paquin D, Beaudin M, (2021). Decreasing Pain and Fear in Medical Procedures with a Pediatric Population (DREAM): A Pilot Randomized Within-Subject Trial. Pain Manage. Nurs 22, 191–197. doi:10.1016/j.pmn.2020.10.002 33495093

[R25] Manne SL, Jacobsen PB, and Redd WH (1992). Assessment of Acute Pediatric Pain: Do Child Self-Report, Parent Ratings, and Nurse Ratings Measure the Same Phenomenon? Pain 48, 45–52. doi:10.1016/0304-3959(92)90130-4 1738574

[R26] McGarry S, Elliott C, Mcdonald A, Valentine J, Wood F, and Girdler S (2014). Paediatric Burns: From the Voice of the Child. Burns 40, 606–615. doi:10.1016/j.burns.2013.08.031 24041516

[R27] McMurtry CM, Riddell RP, Taddio A, Racine N, Asmundson GJ, Noel M, (2015). Far from” Just a Poke”: Common Painful Needle Procedures and the Development of Needle Fear. The Clin. J. pain 31 (10 Suppl. l), S3–S11. doi:10.1097/AJP.0000000000000272 26352920 PMC4900413

[R28] Melzack R, and Wall PD (1965). Pain Mechanisms: A New Theory. Science 150, 971–979. doi:10.1126/science.150.3699.971 5320816

[R29] Miller K, Rodger S, Kipping B, and Kimble RM (2011). A Novel Technology Approach to Pain Management in Children with Burns: A Prospective Randomized Controlled Trial. Burns 37, 395–405. doi:10.1016/j.burns.2010.12.008 21306828

[R30] Newhouse RP, Dearholt SL, Poe SS, Pugh LC, and White KM (2007). Johns Hopkins Nursing Evidence-Based Practice Model and Guidelines. Indianapolis: Sigma Theta Tau International Honor Society of Nursing Indianapolis.

[R31] Page MJ, Mckenzie JE, Bossuyt PM, Boutron I, Hoffmann TC, Mulrow CD, (2021). The PRISMA 2020 Statement: An Updated Guideline for Reporting Systematic Reviews. BMJ 372, n71. doi:10.1136/bmj.n71 33782057 PMC8005924

[R32] Peck TC, Fuchs H, and Whitton MC (2011). “An Evaluation of Navigational Ability Comparing Redirected Free Exploration with Distractors to Walking-in-Place and Joystick Locomotion Interfaces,” in 2011 IEEE Virtual Reality Conference (IEEE), 55–66. 10.1109/VR.2011.5759437 PMC3268068 22297572

[R33] Sahiner NC, and Bal MD (2016). The Effects of Three Different Distraction Methods on Pain and Anxiety in Children. J. Child Health Care 20, 277–285. 26040282 10.1177/1367493515587062

[R34] Scapin S, Echevarría-Guanilo ME, Boeira Fuculo Junior PR, Gonçalves N, Rocha PK, and Coimbra R (2018). Virtual Reality in the Treatment of Burn Patients: A Systematic Review. Burns 44, 1403–1416. doi:10.1016/j.burns.2017.11.002 29395400

[R35] Squires VL (1995). Child-focused Perioperative Education: Helping Children Understand and Cope with Surgery. Semin. Perioper. Nurs 4, 80–87. 7780422

[R36] Witmer BG, and Singer MJ (1998). Measuring Presence in Virtual Environments: A Presence Questionnaire. Presence 7, 225–240. doi:10.1162/105474698565686

[R37] Won AS, Bailey J, Bailenson J, Tataru C, Yoon IA, and Golianu B (2017). Immersive Virtual Reality for Pediatric Pain. Children (Basel) 4, 52. doi:10.3390/children4070052 PMC5532544 28644422

[R38] Xiang H, Shen J, Wheeler KK, Patterson J, Lever K, Armstrong M, (2021). Efficacy of Smartphone Active and Passive Virtual Reality Distraction vs Standard Care on Burn Pain Among Pediatric Patients. JAMA Netw. Open 4, e2112082. doi:10.1001/jamanetworkopen.2021.12082 34152420 PMC8218073

